# Technical feasibility analysis and introduction strategy of the virtually coupled train set concept

**DOI:** 10.1038/s41598-022-08215-y

**Published:** 2022-03-11

**Authors:** Sebastian Stickel, Moritz Schenker, Holger Dittus, Paul Unterhuber, Stefano Canesi, Vincent Riquier, Francisco Parrilla Ayuso, Marion Berbineau, Javier Goikoetxea

**Affiliations:** 1 Institute of Vehicle Concepts, German Aerospace Center (DLR), Rutherfordstraße 2, 12489 Berlin, Germany; 2 Hitachi Rail STS, Genoa, Italy; 3 SYSTRA, Paris, France; 4 Indra, Madrid, Spain; 5 Railenium, Valenciennes, France; 6 Construcciones y Auxiliar de Ferrocarriles, S.A. (CAF), Beasain, Spain

**Keywords:** Environmental impact, Electrical and electronic engineering, Mechanical engineering

## Abstract

Today’s railway network capacity is limited by constraints imposed by traditional train protection systems. A way to overcome those limitations, maximize the railway network performance and also increase the operational flexibility is presented by the Virtually Coupled Train Set (VCTS) concept. This paper evaluates the technical feasibility of this approach, that was developed and is further evaluated in the framework of the Shift2Rail (S2R) project X2Rail-3. The main functionality of virtually coupled train sets is achieved by replacing the mechanical coupler between two railway vehicles by an electronic (virtual) coupling link. This operational change requires a permanent vehicle-to-vehicle communication and precise distance measurement, while enabling much faster coupling and decoupling procedures, increased interoperability and the operation of trains with a headway below absolute braking distance. To evaluate the technical feasibility of the VCTS concept, a series of technical and operational subsystem have been identified and analyzed. Interviews with experts from a variety of VCTS linked topics have been conducted, to evaluate the state of the art and new developments for those subsystems. Subsequently, the capabilities of the subsystems have been compared with the requirements of the VCTS system. In addition, different mitigations to overcome possible obstacles have been identified and evaluated. As the result, the most critical technical aspects for the implementation and success of VCTS have been identified as the requirement of controllable, fast and accurate responding braking systems, the availability of suitable communication technologies and frequency bands, the need for highly-accurate measurement of distance, speed and acceleration and the fast detection and monitoring of train integrity. Considering those results, a qualitative roadmap for the future VCTS development and introduction strategy is derived.

## Introduction

### Background and motivation

With an increasing demand in passenger and freight transportation, railway networks are approaching their capacity limit, especially in densely populated areas and on highly frequented lines. This leads to a lack of flexibility within the railway operation, resulting in delays and overcrowding for passengers or the lack of transportation capacities in the case of freight transportation. An expansion of the railway infrastructure is not always possible due to the lack of space for additional rails, platforms or stations. In addition, new infrastructure is very cost-intensive, while the planning, permission and building takes a long time. Virtual coupling of train sets presents a viable solution to resolve the presented problems. The aim is to increase the track capacity by decreasing the distance between trains. While the development and implementation of the European Train Control System (ETCS) Level 2 (radio based fixed block approach) and ETCS Level 3 (moving block approach) can present a substantial increase of railway capacity already, the Virtually Coupled Train Set (VCTS) paradigm goes beyond those concepts. By establishing a continuous communication between the trains and utilizing a cooperative braking curve, those vehicles can be operated in relative braking distance, as opposed to absolute braking distance. This allows for an even lower separation between trains, further increasing track capacity.

While the concept of virtually coupled train formations^1^ or virtual train-sets^2^ has been proposed for many years, research has been increased recently. VCTS concept descriptions have been presented by Goikoetxea^3^, Winter^4^ and Flammini et al.^5^, while an extensive investigation of the virtual coupling concept was agreed on in the Shift2Rail Master Plan^6^, allocated in the X2Rail-3 project. Different publications have been dedicated to possible VCTS control and operation scenarios^7–10^, the comparative and numerical analysis and simulation of operational benefits^11–15^ as well as safety related issues^16,17^. Other research was focussing on specific technological enablers of VCTS^18,19^ or Automatic Train Coupling^20^. However, the technical feasibility of the VCTS concept is analysed for the first time in such detail in this publication.

Results of this feasibility analysis have been obtained within X2Rail-3, which is a Horizon 2020 project of the Shift2Rail Joint Undertaking, focussing on the “Advanced Signalling, Automation and Communication System” for railway operation in order to promote capacity increase, automation and flexible communication^21^. Within this framework, the VCTS concept was developed, specified and analysed^22^. Next to the general feasibility of the VCTS concept, special focus of those investigations are application conditions, the performance and safety of the system, the functional and non-functional architecture and requirements as well as the impact on the existing railway systems and a possible business model.

### Aims and scope

The aim of this investigation is to assess the general feasibility of the developed VCTS concept. This includes the analysis of the applicability of essential enablers (technologies and interfaces) to facilitate VCTS operation, e.g. novel communication technologies. The technical and operational capabilities and limitations of those enablers are compared to the qualitative requirements of the VCTS system in order to evaluate their feasibility. Potential limitations or obstacles are evaluated in terms of criticality, while mitigation measures are proposed. Those solutions can be of technical or operational nature. In addition to specific technological challenges, a number of general obstacles are identified, stemming from the conventional railway signalling paradigm or the implementation of a novel concept into an established environment. Moreover, recommendations for possible VCTS implementation pathways are derived from the feasibility analysis, to foster the introduction of the system into current railway operation. In addition to the technical feasibility of the proposed concept, assuring interoperability of VCTS with current signalling system approaches is essential and therefore is elaborated upon.

## VCTS system concept description

**Figure 1:**
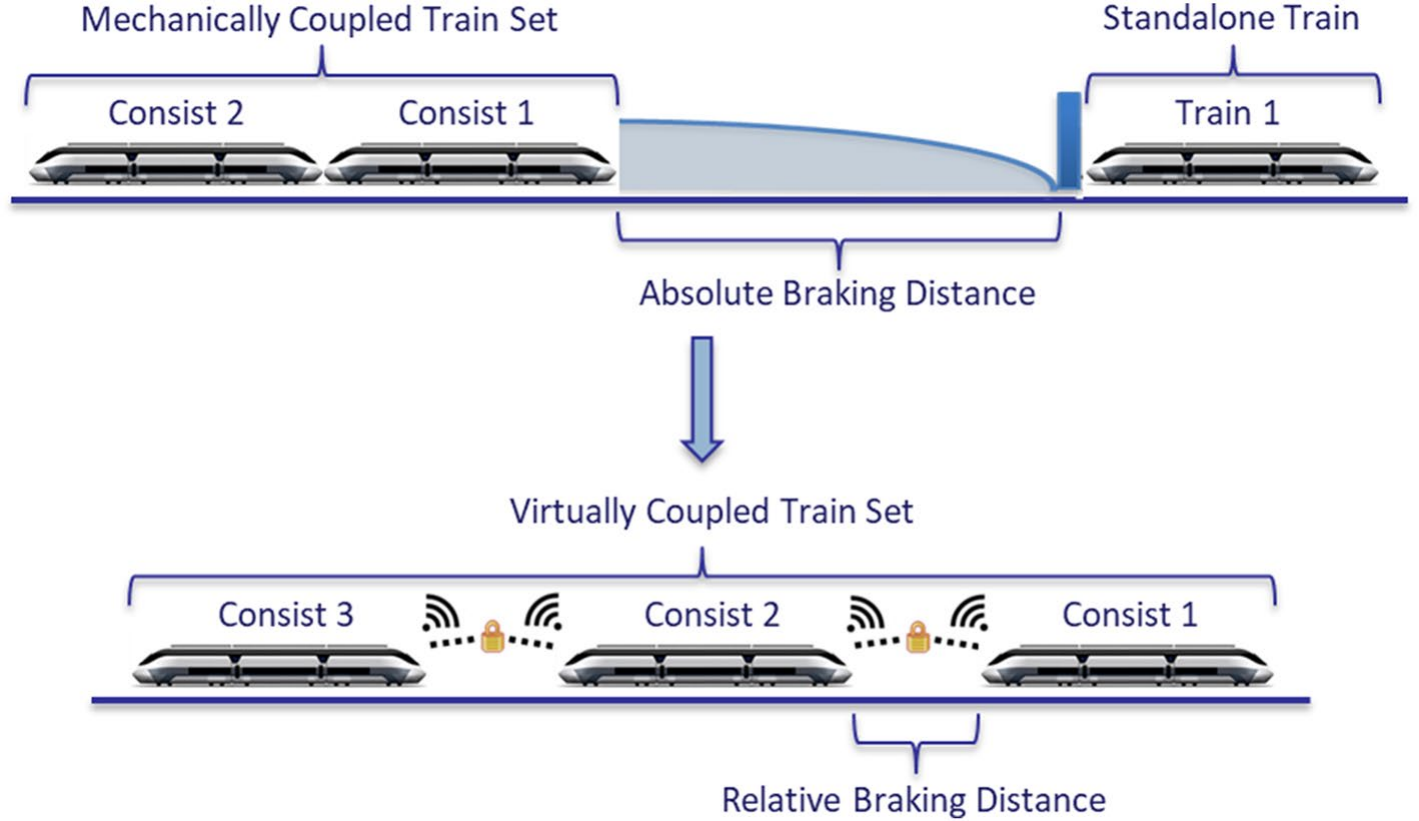
Overview of mechanically and virtually coupled train set paradigms and the shift from absolute to relative braking distance, exemplary representation as distances are larger in reality.

Conventionally, train sets are based on a mechanical coupler that connects the consists of a train set with each other and transfers force, information and pressure (for pneumatic braking systems) from one unit to the next one. The basic function of the mechanical link is therefore to keep the relative position between the coupled units fixed, while exchanging information and possibly energy. In this coupling paradigm, train sets operate in absolute braking distance as shown in the upper part of Fig. 1. The concept of VCTS is centred around the replacement of this mechanical coupler by a virtual link which is based on a continuous, reliable and secure exchange of information between all units within a train set. The absence of the physical connection indicates that the single units of a train set may have, at any time, different kinematic behaviours. As a result, the main function of the virtual coupling is the maintenance of a coordinated and safe distance between the single units, the cooperative train headway, while enabling driving in relative braking distance as opposed to absolute braking distance (Fig. 1, bottom part). To achieve this coordinated headway between the single units of a train set, each unit needs to compute, manage and communicate the headway based on its own specific acceleration- and braking characteristics, the characteristics and capabilities of other virtually coupled units, as well as the current dynamic information, e.g. speed and position, of all units. The longitudinal controller is of major importance to ensure string stability of the platoon. While the implementation of the control algorithm was not part of the feasibility analysis, different approaches and solutions have been discussed in the related workstream and the scientific society. String instabilities can be expected if only the relative spacing information is utilized to control distance to a or preceding vehicle^23,23^. The VCTS concept aims for communication between all vehicles of a platoon, merging the benefits of different control architectures, such as multiple predecessor following and leader and predecessor following, to guarantee string stability^25,26^. Thus, the longitudinal controller is dependent on the headway and speed of the vehicles within the platoon and the latency and reliability of the communication system.

In order to ensure at least the same level of safety as in current railway operation, the VCTS components (e.g. sensors, communication) are designed as a redundant and fail-safe system, providing the necessary safety integrity levels (SIL) across all system levels. Whilst designing the VCTS concept, an exhaustive operational safety and functional hazard analysis has been conducted, presenting safety requirements for the VCTS operation^27^. Furthermore, the operating principle of VCTS is following a positive train control approach: If a message between two vehicles is not received or answered within a set frame, or if communication is lost, appropriate safety measures will be taken. VCTS will also utilize safety measures implemented in the underlying signalling system, such as automated train protection or the traffic management system, to interact with non-VCTS vehicles.

**Figure 2:**
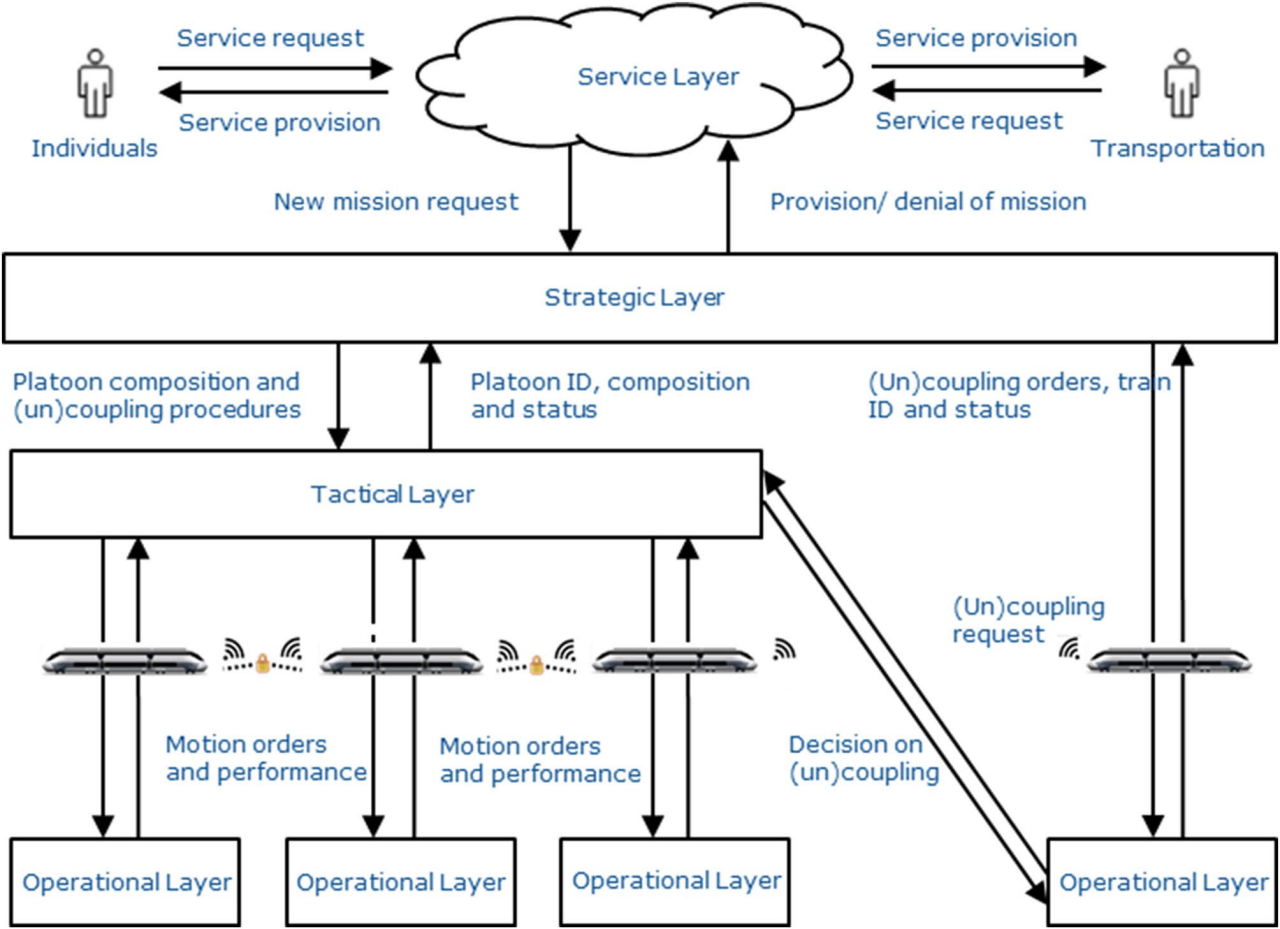
VCTS functional layers based on^22^.

On a functional level, the VCTS system can be decomposed into four layers, interacting with one another or external actors. A schematic overview is given in Fig. 2. The service layer coordinates the need for services as well as the interaction between service and user. The information is distributed to the strategic layer, which determines the ideal utilization of available railway vehicles. Its main objective is therefore to maximize the capacity of the infrastructure while supervising traffic flow. The actual movements of the virtually coupled units are coordinated by the tactical layer, usually governed by the master train. It is responsible for the definition and supervision of the coordinated train headway and manages unexpected events (e.g. degraded situations). The operational layer is in charge of the local control (e.g. accelerating and braking) of each consist, assuring that commands established by the tactical layer are safely executed.

Considering these definitions, five core VCTS functions can be identified. Those are the virtual coupling set up, the coupled driving (coordinated platoon movement), the termination of the virtual coupling, the interaction with external systems and finally, the protection of the consists inside the VCTS from collision. All of those functions are implemented by a combination of different technical and operational subsystems like suitable braking systems, communication technologies or train integrity monitoring concepts. The layered structure allows VCTS to be implemented as a complimentary system. It operates through interfaces to the underlying signalling and train protection system and can therefore be utilized in any existing or future railway network.

The VCTS concept offers a wide range of potential benefits when compared to other signalling and train protection paradigms^11–15,28^. The benefits can be roughly categorized into operational or monetary benefits and are expected to result in an increased competitiveness in railway freight and passenger transportation. Table 1 gives a qualitative summary of those potentials.

**Table 1:** Potential benefits of VCTS over conventional signalling.

Category	Benefit
Operational and monetary	Increased line capacity
Operational	More robust schedule
Operational and monetary	Less time- and labor-intensive coupling/ decoupling
Operational	Dynamic modification of train set composition while driving
Operational	Interoperability between different vehicle types, models and manufacturers
Operational	Maximized utilization of infrastructure (e.g. platforms and stations)
Operational	Optimization of new mobility concepts (e.g. rail-taxi, demand-based mobility)
Monetary	Reduced global investment-, maintenance- and operational costs by implementation of on-board systems
Monetary	Potential omission of side tracks and railyards for manual coupling

As the developed VCTS concept is highly complex, it is reasonable to schedule introduction and implementation in incremental steps. However, a number of minimum requirements must be achieved, in order to make the introduction of this new paradigm viable from an operational, economical and safety related standpoint. Those minimum requirements are:

- Enabling driving below absolute braking distance to increase track capacity
- Reduced time and effort for de-/coupling procedures compared to mechanical coupling
- Operation at the same or higher safety level as in current operation
- Provision of compatibility with existing infrastructure and independence from signalling system

## Methodology

**Figure 3:**
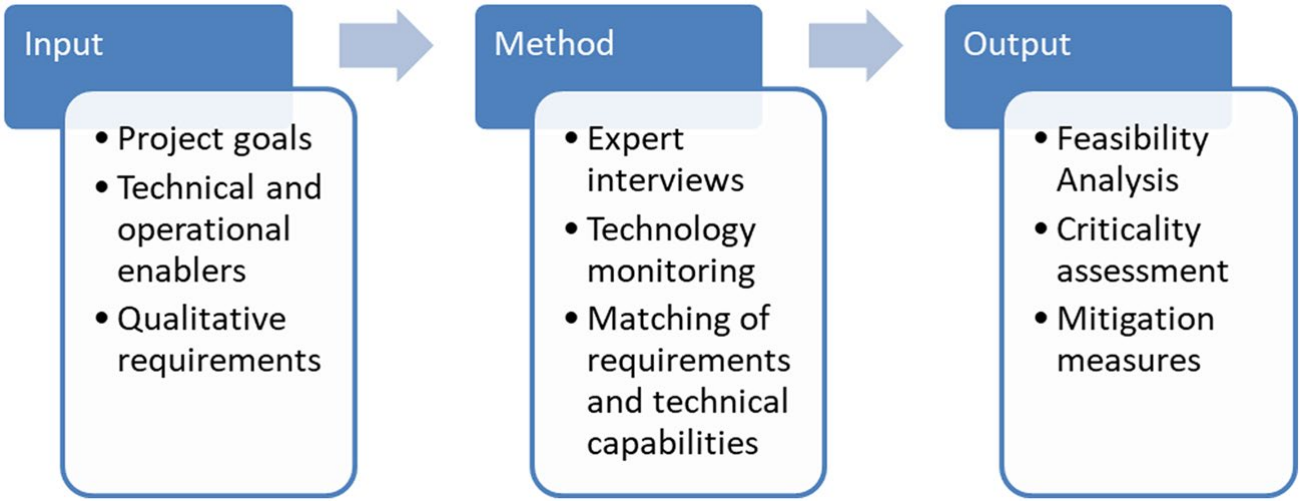
Methodical approach to feasibility analysis, based on^29^.

To evaluate the feasibility of the proposed VCTS concept, DIN 69901-2-D.8.3^29^ was used as a reference. This norm gives a demonstrative overview of necessary inputs and possible methods that can serve as indications for the feasibility analysis of a given project. It was adapted to fit the goal of this analysis as shown in Fig. 3. The methodological approach of this analysis is centred on the consideration of novel technical and operational solutions that have been investigated in related work streams in the X2Rail projects and the Shift2Rail Joint Undertaking. By utilization of these already acquired insights, state-of-the-art technology as well as future developments regarding necessary technologies and procedures can be integrated into the feasibility study. The main component of the feasibility analysis is to match the requirements of the VCTS concept with the current and projected capabilities of the related technical components and operational procedures.

### Identification of technical and operational enablers and interfaces

The virtual coupling of trains is based upon the connection and interaction of different subsystems, including technical components and operational procedures, subsequently summarized as enablers. The identification of those relevant subsystems is the basis of the presented analysis. Each enabler can be linked to qualitative requirements that need to be fulfilled in order to achieve full VCTS functionality. However, not all requirements need to be implemented to enable core VCTS operation, as elaborated upon in the introduction strategy section of this report. An overview of this correlation is given in Table 2. The identification of all relevant enablers was a main focus of the precedent VCTS concept development and is based on the experience and expertise of all contributing project partners, an extensive literature review and technology scoping and discussions with external experts.

**Table 2:** Qualitative requirements for VCTS enablers to facilitate basic VCTS operation.

Enabler	Qualitative requirement
Brakes and traction	VCTS should be able to control traction and brakes of all consists
	Modulation of the SIL 4 brake system to manage cooperative braking behaviour, short build-up times
	Calculation of highly accurate brake characteristics to reduce safety margins due to uncertainties
	Low adhesion and brake failure management
	Availability of real-time information of all brake and traction parameters
	Availability of real-time track data
Communications	Communication (T2T and T2G) following RAMS and security principles
	Unique identification of message source and destination, timestamping/sequencing, status monitoring
	Provision of high information quality and distribution rates in correspondence to the headway
	Utilization of communication sequence protocols for certain actions/manoeuvres (e.g. coupling and decoupling)
Field elements (e. g. balises, switches, level crossings) and their management	Updated functions to allow virtually coupled platoons in single block sections and to pass field elements without interference
	Availability of sufficiently low switching times and status update communication
	Allowance of VCTS manoeuvres (e.g. de-/coupling via switch)
Interoperability with existing signaling systems	VCTS shall be independent of the signalling systems which is active on the utilized network
	The signalling and the connected interlocking and ATP shall not inhibit VCTS operation
	Bi-directional communication between the VCTS platoon and the trackside system (Strategic Layer, TMS)
Platforms and stations	Appropriate handling of increased vehicle and passenger throughput
	Availability of sufficient space for platoons and passengers
Traffic management system	Implementation of algorithms and functionality to enable VCTS operation and maneuvers
Train integrity	Continuous information of train integrity by on-board solutions; immediate and safe response in case of train integrity loss
Train operation: MO, ATP and ATO	Interface to VCTS to set up a platoon, run diagnostics and maintenance
	Availability of both automated and manual control of the leading consist
	ATP and ATO compatibility with VCTS via interface or direct implementation
Train positioning	Provision of highly accurate relative and absolute distance, speed and acceleration and real-time track-data

### Expert interviews

For each relevant technological and operational subsystem, interviews with respective experts were performed to evaluate the state-of-the-art technologies as well as ongoing developments and their ability to provide VCTS functionalities. For the interview process, a general questionnaire of 55 questions was defined, covering a wide range of VCTS related topics. The questionnaire was then adapted to specifically address relevant points of interest for each subsystem, resulting in a total of 145 questions. The questionnaire was then distributed to 12 working groups from the Shift2Rail framework, each working on a related subsystem, therefore commanding specific expertise. All contacted working groups were available for discussions and interviews. The questions were discussed within each group, overall representing 150 experts, leading to interviews with 20 different representatives. After one review loop, the consolidated answers have been utilized to propose solution strategies for possible obstacles. Those mitigation measures have also been evaluated by the experts in a second review loop. Based on those results, the criticality of the possible utilization of the subsystems for the VCTS introduction has been evaluated. This evaluation was presented to the experts for a final review loop.

### Informed consent

All interview-participants agreed, that the results of the interviews will be evaluated and utilized in project reports and further dissemination activities. Informed consent was therefore obtained.

## Results: identified critical aspects for VCTS enablers

As the developed VCTS concept necessitates several requirements on various technical and operational enablers of the railway system, these requirements may present significant challenges for today’s standards. After evaluation of the state of the art and current developments, the ability of those enablers to cover the requirements for VCTS operation has been derived. The thereby identified critical aspects, potential obstacles and different mitigation measures for the introduction of VCTS are summarized in Table 3.

**Table 3:** Identified critical aspects with regard to VCTS and suggested mitigation measures.

Enabler	Critical aspect	Possible solution/mitigation measure
Brake system	Performance of current SIL4 braking systems insufficient for safe VCTS headway management	Development of SIL4 electronic braking system with improved modulation, accuracy and response time, utilization of closed loop control
	Handling of low wheel-rail adhesion and brake failures	Utilization of real-time adhesion measurement, permanent brake monitoring and closed-loop brake control. Increased information accuracy to optimize safety margins
Communications	Low latency requirements not achievable with current technology	Latency reduction with T2T communications or simplified fixed network structure. Low latency features promised by future V2X communication standards (e.g. IEEE 802.11bd)
	Headway between high speed and freight trains requires long communication ranges	Increased transmission power or utilization of MIMO transmission to extend communication range. Simultaneous operation of different technologies to increase frequency specificity
	Increased retransmission data rates required, if threshold for the maximal tolerable packet error rate is exceeded	Increased transmission power or retransmission rates, utilization of MIMO transmission or error code correction by combination of several technologies
	Availability (marked readiness) of rail certified communication systems	Usage of adaptable communication systems (ACS): achieving redundancy by combination of different systems results in better coverage or optimized dimensioning to support high traffic and coverage
	Limited availability of spectrum bands	Utilization of license free spectrum bands (only reasonable in mmWave bands); shared ITS-band (5.9 GHz) usage for railway and road applications; acquisition of license for new railway exclusive bands; consideration of cognitive radio approach with primary and secondary user
Field elements	Current switching technology not suitable for demanding VCTS operation	Development of fast point machines with instant status notification in order to allow general VCTS operation and coupling/de-coupling procedures
	Level crossing	Technology adaptation to account for multiple consists along the level crossing
Interoperability with existing signaling systems	New coupling scenarios and variable platoon length can be conflicting with current interlocking paradigm	Introduction of new communication protocols and software-based implementation of new scenarios into interlocking rules
	Presence of two non-physically coupled trains in one section not foreseen in current railway signaling	Adaption of the ATP logic regarding VCTS functionality to prevent stopping of virtually coupled trains
Platforms	Space restrictions at existing stations	Utilization of VC procedures (calling at multiple platforms); adaption of passenger steering to faster platform clearance; optimization or development of new platform layouts
Traffic management system	Functional architecture of VCTS and TMS interactions undefined	Software based definition of VCTS-TMS interactions
Train integrity	Currently long detection times for loss of train integrity	Novel solutions required to provide sufficiently low train integrity loss detection times
Train operation: MO, ATP and ATO	Slow reaction times in manual operation	Sufficient safety margins, operation in ATO mode where available
	Interaction of ATO and ATP with VCTS undefined	Definition of VCTS interaction, software-based implementation
	Increasing complexity of on-board traction/brake control interfaces	Utilization of available interfaces or development of novel VCTS interface
Train positioning	Current technology not accurate enough	Development of high accuracy train positioning solutions complemented by suitable redundant distance measuring

## Discussion

### Assessment of criticality

**Figure 4:**
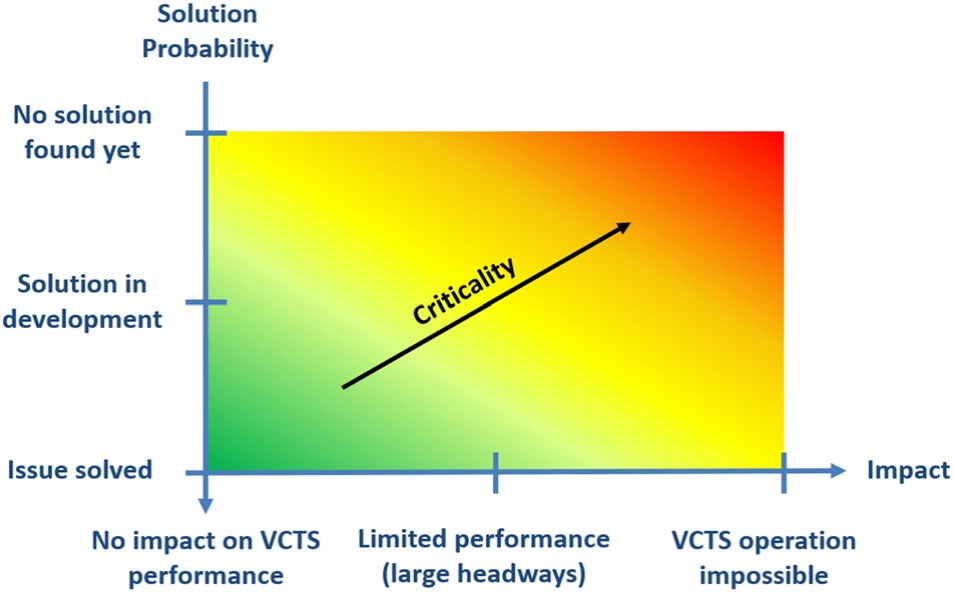
Criticality matrix, qualitative approach to evaluate obstacles for the VCTS introduction.

All of the aforementioned enablers are important for a successful and well-performing VCTS system, but not equally critical regarding VCTS implementation and operation. Criticality is usually assessed as a combination of severity and likelihood of occurrence when referred to the risk of failure (e.g. see EN 50126^30^). To fit the scope of this analysis, this methodology was adapted, redefining the indicators of the criticality as shown in Table 4. By merging the indicators into a chart, a criticality matrix (Fig. 4) was created. Utilizing the results of the expert interviews and the preliminary analyses, the criticality of all identified enablers was assessed. As the requirements on the VCTS enablers can vary for different railway market segments, the criticality assessment has been carried out for 5 base scenarios; high speed, regional, metro, tram/light rail and freight.

**Table 4:** Adapted definition of criticality indicators.

Indicator	Adapted definition
Severity	Negative impact of today’s technology or procedures on the VCTS performance
Likelihood	Probability, that the issue is not solved in the foreseeable future

Following this methodology, some of the defined enablers, namely train operation, platforms and TMS, can be evaluated as not critical regarding VCTS implementation and operation. The applicable solutions (see Table 3) are either available or can be realized without further necessary research. The remaining enablers present more pressing obstacles. Those aspects are further elaborated upon in the following:

#### Brakes

In the VCTS concept, it is foreseen that the braking effort of the consists should be precisely adaptable with respect to the capabilities of the tailing consists to ensure cooperative braking behaviour. The current achievable performance of the pneumatic system, utilized in the emergency brake, does not fulfill this requirement. While some suitable service braking systems already utilize electronic control chains and provide the necessary performance, they do not meet the VCTS requirement of SIL4. This sets the brake system to the right of the criticality matrix, as VCTS is heavily limited by additional safety margins to guarantee safe braking manoeuvres with non-modulable braking efforts. The minimum possible headway additionally increases with respective margins for long brake force build-up times and low accuracies of the stopping distances, especially pronounced in freight traffic. Depending on the specific vehicle characteristics, this might endanger the aim to operate VCTS below absolute braking distances and thus lose potential benefits when compared to a Moving Block system. A possible solution to this issue is the introduction of SIL4 electronic brakes and closed-loop controls. While these systems are already utilized with a lower safety level for service braking, developments for emergency brake systems are already ongoing. The issue is thus expected to be solved within the next years. The handling of low wheel-rail adhesion was also identified as critical. Here, real time adhesion measurement, permanent brake supervision and the monitoring and communication of rail conditions could present feasible solutions in the future.

#### Communications

Potential obstacles regarding communication technologies vary significantly between different market segments. The related variation of service velocities, and thus differing relative braking distances, sets different requirements for communication range and latency. While for high speed trains the communication has to ensure very fast message delivery over a rather long range (hundreds of meters up to several kilometres), lower speed applications, such as metro or tram, operate with much shorter distances for similar headways. To cover this wide range of requirements, the application of adaptable communication systems (ACS) and the introduction of next generation communication technologies has been discussed. Another market segment overlapping obstacle is the availability of frequency bands for railway applications. The issue remains subject to further political decisions regarding additional allocation of frequency bands or needs to be mitigated with workarounds like spectrum sharing or a cognitive radio approach. As no exact time horizon for the resolution of those issues can be identified, the communication aspect is categorised as medium to high criticality. The introduction of new communication protocols was evaluated as demanding but not critical. By utilizing a mobile communication standard which supports future railway mobile communication system (FRMCS) requirements, the process can be facilitated. Those standards will be specified by IEEE or 3GPP and refined by the relevant protocols, e. g. 802.11p, 802.11bd or R15 LTE-V2X.

#### Field elements

Field elements and their management were also identified as a critical aspect regarding the implementation of VCTS. In particular, to enable safe and fast coupling and decoupling maneuvers, it has to be ensured that switches can change their positions very quickly and in a fail-safe way, while also sending instant status updates to the platoon, infrastructure- and traffic management. Current developments deal with switches capable to reach those performance requirements. This issue applies to all railway scenarios and can be located in the middle of the criticality matrix as it sets limits to the overall VCTS performance. The technological solutions are expected to be available in the near future.

#### Interoperability

Closely connected to the field element management is the handling of VCTS by existing signaling systems. To the signaling, the platoon (also during coupling or decoupling) has to appear as only one train, analogous to a mechanically coupled train, but with a variable length. The current coupling status needs to be communicated permanently to the underlying signaling-, traffic management- and train protection system. Those systems should not interact with the single consists of a VCTS but with the whole platoon. The individual consists do not follow the signaling logic as the distance and safety is controlled by the VCTS master consist. As a result, the whole VCTS follows the same signaling logic, representing a single train. It is viable that the necessary adaptions, in terms of software- and signaling logic, like blocking all sections occupied by the VCTS, can be implemented. Although not without challenges, the introduction of those new rules is expected to align with interests of the industry and railway operators, facilitating this implementation. The described issues can be placed in the lower area of the criticality matrix. If VCTS is to be used as a secondary system alongside conventionally coupled train operation, interoperability is naturally ensured by the underlying signaling system.

#### Train positioning and sensors

The accurate measurement of distance as well as relative speed and acceleration between the consists is implicitly critical for VCTS operation. The distance regulation relies on highly accurate state measurements, incorporating a SIL4 approach. Current developments in absolute positioning aim for a high accuracy with 2% error margin, with respect to the last reference point. Additional improvements for relative measurements can be achieved by the application of suitable and redundant sensor combinations, such as RADAR, LIDAR, etc. Further improvements in vehicle positioning are expected by the utilization real time track data, digital maps or a virtual balise approach. The impact of vehicle positioning and distance measuring accuracies on the VCTS performance depends mainly on the speed level and therefore the railway market segment. The higher the service velocity the more critical the issue becomes. Assuming the availability of those much more accurate and faster train positioning measurements, a headway reduction between 64 and 81% can be achieved, depending on the railway scenario and reference case^31^^32^. With the assumption of certain technology goals and boundary conditions, this translates to a headway of 90 m for metro or subway applications, 300 m for regional services, 550 m in freight applications and 860 m for 300 km/h high speed services^31^. All values include reaction delays, distance and speed measurement inaccuracies and an additional safety margin.

#### Train integrity

On-board train integrity detection is a crucial aspect of VCTS operation. In order to achieve distances below absolute braking distance, a very frequent update on the train integrity needs to be achieved. The current aim of 5 s^33^ for wireless detection systems would cause a large safety margin on top of all other inaccuracies, significantly limiting the VCTS performance (mid-right in the criticality matrix). Possible solutions include TI detection based on novel wireless sensor networks, as also utilized for train positioning measurements, or satellite-based solutions. This is mainly an issue for cargo trains, due to the long non-fixed formation. Passenger trains may also utilize wired on-board train integrity solutions, which aim for a performance of 1 s for integrity loss detection. Platoon integrity monitoring will be established by T2T communication based on protocols to be defined in future VCTS works.

**Figure 5:**
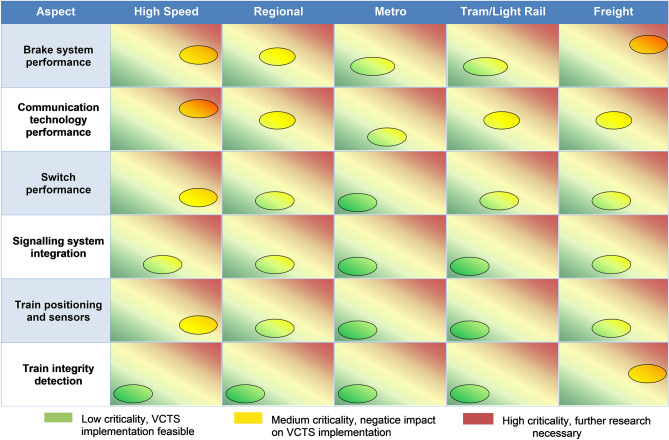
Qualitative VCTS criticality assessment of the most important identified aspects, based on expert interviews.

A graphical interpretation of the abovementioned aspects, with respect to the criticality matrix, is given in Fig. 5. It can be concluded that metro and light rail applications are facing the lowest amount of potential critical obstacles regarding VCTS implementation. While the number of aspects with higher criticality is increasing for regional applications, the most obstacles apply in particular to freight and high-speed transportation.

### VCTS introduction strategy

Based on the presented evaluation of the general feasibility, an introduction approach for VCTS was derived. To limit the complexity and amount of simultaneous technical and operational changes, an incremental implementation should be targeted. The approach is divided into two main stages.

#### Stage 1 (VCTS core functionality/operation)

**Figure 6:**
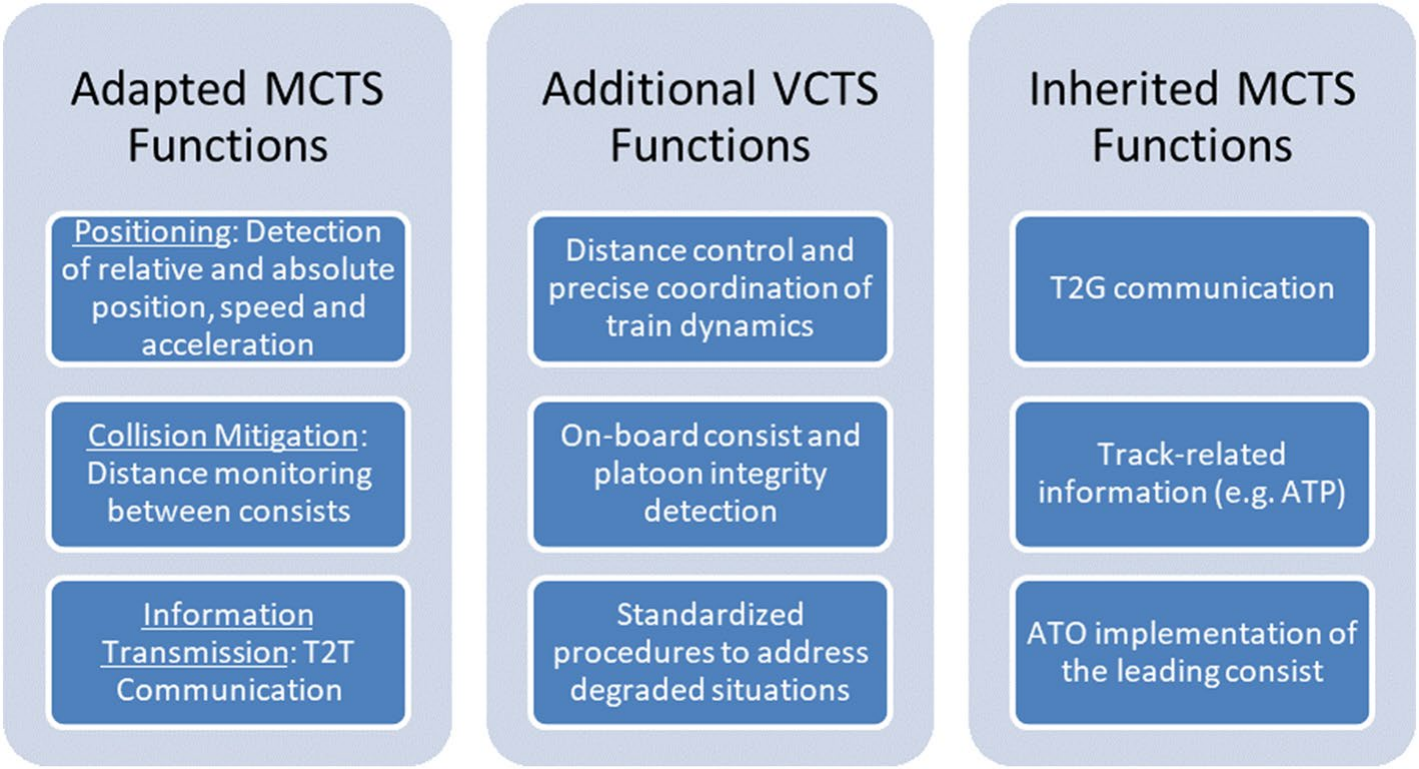
Functions to be provided by VCTS in stage 1.

In stage 1, the mechanical coupling between two or more consists will be replaced by virtual links, enabling basic VCTS operation. However, coupling is still done while in standstill. All functions that are provided or distributed by the mechanical link need to be transferred to a digital system, while additional functions of the virtual coupling concept have to be included. The relevant functions can be split into three groups as shown in Fig. 6. A significant increase in operational flexibility can be achieved with an extension of stage 1. As the mechanical link is replaced by a digital one, there are no restrictions for coupling of different vehicles. This will enable coupled operation with different vehicle types.

#### Stage 2 (additional functional modules to achieve full VCTS functionality)

Stage 2, aims to provide additional functionalities to the VCTS core operation introduced in stage 1. These functionalities aim to further materialize the benefits of the VCTS concept. Stage 2 represents a pool of functional modules that can be added simultaneously or successively, depending on the operational needs and technical possibilities. Possible modules include coupling and/ or decoupling on the fly or via switch and implementation of additional manoeuvres (e.g. call at different platforms simultaneously). This new dynamic approach allows for coupling and decoupling according to needs of live operation, mitigating or even preventing potential operational delays, by utilizing new TMS functions.

Considering the results of the criticality assessment, it can be concluded that the VCTS implementation will face less challenges in low-speed and non-freight applications. This conclusion is reinforced by further market segment characteristics which should facilitate the VCTS implementation. Those may include closed network structure, uniform vehicles, the lack of interaction with other vehicle types or level crossings and reduced aerodynamic hazards or environmental effects, among others. Therefore, it can be concluded that urban railway scenarios such as metro or subway present the most feasible application for the early stages of VCTS implementation, followed by lower speed regional services.

### VCTS implementation roadmap

**Figure 7:**
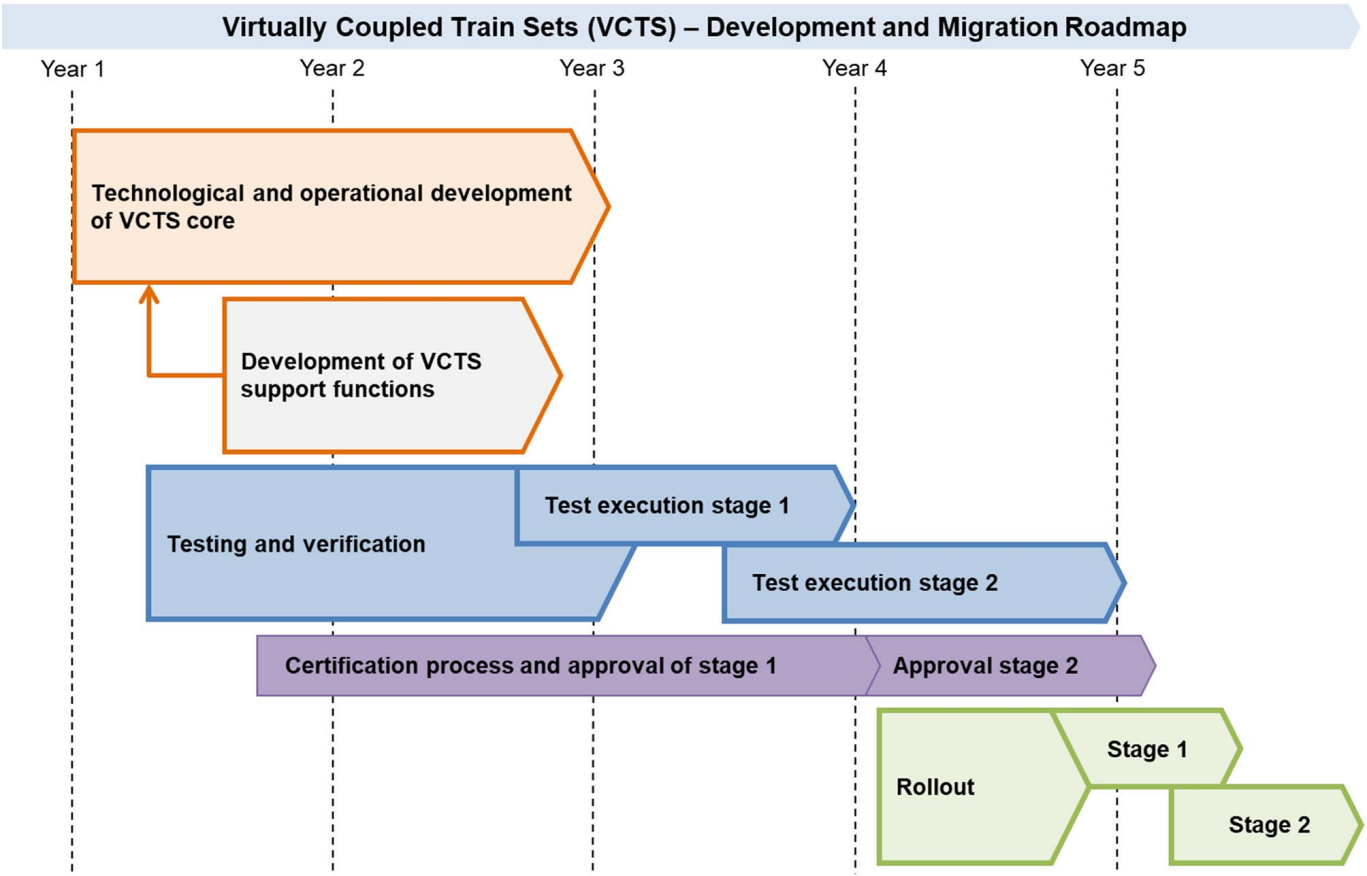
Qualitative roadmap for VCTS implementation.

Following the results of the feasibility analysis, a general development and migration roadmap for the VTCS concept can be derived. The first step includes the development of VCTS core functions and technologies, that are specific to VCTS operation (stage 1). This includes the specification and development of sensors, control and communication protocols. The development of support functions is not specific to VCTS itself and will be beneficial to conventional railway operations as well. Examples are modern braking systems, T2G communication systems or cyber security technology. Testing and verification involves the development of testing concepts infrastructure and finally VCTS demonstrators. These processes and especially the following test execution run in parallel to the technological and operational development and should interact with each other in feedback loops. Accompanying both the development and testing is an ongoing certification process of the concept and its subsystems. After the approval of VCTS core operation (stage 1), testing will still be ongoing for the additional functional modules (stage 2). Upon rail approval, VCTS core operation can be introduced. The order of implementation of additional functional modules of depends on the progress of VCTS development as well as on the preferences of railway operators with suitable networks. This roadmap is visualized in Fig. 7. The chosen timeframe is based in the feasibility evaluation but can vary based on further developments. While a basic VCTS functionality testing has been carried out by CAF^34^ and the Russian Railway^35^ on isolated test sites, more VCTS research, development and on-track testing is expected to originate from the Shift2Rail successor Europe’s Rail Joint Undertaking^36^.

## Conclusions

This paper summarizes the analysis of the technical feasibility of the VCTS concept. VCTS itself can be divided into a range of technological and operational enablers and related external components. Qualitative requirements of the VCTS concept have been matched with the current and projected capabilities of those enablers to evaluate their applicability in VCTS operation. Based on the presented approach, critical aspects for a successful VCTS implementation have been identified. The most critical issues are the need for highly accurate measurements of distance, relative speed and relative acceleration and the requirement of modulable, fast and accurate responding braking systems. Additionally, the availability of both communication technologies and dedicated railway frequency spectrums, paired with safety requirements for latency in combination with long communication ranges and the requirement of fast and reliable detection of train- or consist integrity loss by on-board equipment are similarly critical. For all identified critical aspects, technological or operational solutions or mitigation measures have been presented. Regarding the analysis, aspects have been found to be more critical in high-speed and freight applications. The VCTS introduction approach has been divided into two distinct stages to facilitate the implementation. Stage 1 aims to substitute the mechanical coupler with the virtual link, while stage 2 adds additional functionalities to the VCTS concept. Following this proof of feasibility of the concept, a qualitative development and migration roadmap was proposed.

## Data Availability

The X2Rail-3 deliverable that this paper is based on is not publicly available. However, previous deliverables, detailing the VCTS system concept as well as a performance analysis, are available at the X2Rail-3 online portal^22^.

## Abbreviations

ACS: Adaptable communication systems
ATO: Automatic train operation
ATP: Automated train protection
ETCS: European train control system
FRMCS: Future railway mobile communication system
MIMO: Multiple input multiple output
RAMS: Reliability, availability, maintainability, safety
S2R: Shift2Rail
SIL: Safety integrity level
T2G: Train to ground
T2T: Train to train
TMS: Traffic management system
VCTS: Virtually coupled train set
